# Established Tumour Biomarkers Predict Cardiovascular Events and Mortality in the General Population

**DOI:** 10.3389/fcvm.2021.753885

**Published:** 2021-12-08

**Authors:** Valentina Bracun, Navin Suthahar, Canxia Shi, Sanne de Wit, Wouter C. Meijers, IJsbrand T. Klip, Rudolf A. de Boer, Joseph Pierre Aboumsallem

**Affiliations:** Department of Cardiology, University Medical Center Groningen, Groningen, Netherlands

**Keywords:** onco-cardiology, cardiovascular disease, biomarkers, tumour, heart failure, cardiotoxicity

## Abstract

**Introduction:** Several lines of evidence reveal that cardiovascular disease (CVD) and cancer share similar common pathological milieus. The prevalence of the two diseases is growing as the population ages and the burden of shared risk factors increases. In this respect, we hypothesise that tumour biomarkers can be potential predictors of CVD outcomes in the general population.

**Methods:** We measured six tumour biomarkers (AFP, CA125, CA15-3, CA19-9, CEA and CYFRA 21-1) and determined their predictive value for CVD in the Prevention of Renal and Vascular End-stage Disease (PREVEND) study. A total of 8,592 subjects were enrolled in the study.

**Results:** The levels of CEA significantly predicted CV morbidity and mortality, with hazard ratios (HRs) of HR 1.28 (95% CI 1.08–1.53), respectively. Two biomarkers (CA15-3 and CEA) showed statistical significance in predicting all-cause mortality, with HRs 1.58 (95% CI 1.18–2.12) and HR 1.60 (95% CI 1.30–1.96), when adjusted for shared risk factors and prevalent CVD. Furthermore, biomarkers seem to be sex specific. CYFRA 21-1 presented as an independent predictor of CV morbidity and mortality in female, but not in male gender, with HR 1.82 (95% CI 1.40–2.35). When it comes to all-cause mortality, both CYFRA and CEA show statistical significance in male gender, with HR 1.64 (95% CI 1.28–3.12) and HR 1.55 (95% CI 1.18–2.02), while only CEA showed statistical significance in female gender, with HR 1.64 (95% CI 1.20–2.24). Lastly, CA15-3 and CEA strongly predicted CV mortality with HR 3.01 (95% CI 1.70–5.32) and HR 1.82 (95% CI 1.30–2.56). On another hand, CA 15-3 also presented as an independent predictor of heart failure (HF) with HR 1.67 (95% CI 1.15–2.42).

**Conclusion:** Several tumour biomarkers demonstrated independent prognostic value for CV events and all-cause mortality in a large cohort from the general population. These findings support the notion that CVD and cancer are associated with similar pathological milieus.

## Introduction

Cardiovascular disease (CVD) and cancer are the leading causes of mortality worldwide (1). Therapeutic advances, despite improving survivorship, have increased the overlap between these two syndromes. Now, millions of cancer survivors have an increased risk for CVD, either from shared lifestyle risk factors, or from toxicities of cancer treatment. Therefore, cardiac care of cancer survivors is essential (2). On the other hand, many studies hinted toward an association between CVD and cancer incidence and mortality (3, 4). Furthermore, a wealth of clinical and preclinical data supports the bidirectional relation between CVD and cancer (5–8).

Shared biological mechanisms and risk factors explain the link between CVD and cancer. Among others, pathways related to inflammation, clonal haematopoiesis, hypoxia, and circulating factors are of interest in this regard (9). Further, shared risk factors such as smoking, obesity, hypertension and lack of physical activity are recognised as common initiators of both syndromes (10). Recent data show that CVD has a high impact on long-term morbidity and mortality in patients with newly diagnosed cancer and cancer survivors (3, 11). These outcomes are only partially explained by short and long-term complications due to cardio-toxic treatments (12). Structural changes in the heart and elevation of cardiac biomarkers have been reported in patients with cancer even before the initiation of any antineoplastic treatment (13).

Our group has previously shown that heart failure (HF) directly promotes intestinal tumour growth (7). These findings were further supported by another preclinical study showing early cardiac remodelling preceding HF promotes breast cancer (8). Nonetheless, the direct pathophysiological mechanisms linking those two entities are still unknown.

The notion that cancer and CVD are two entities of the same syndrome was further supported by our most recent study showing that a number of tumour biomarkers, measured in patients with prevalent HF, can predict HF severity and all-cause mortality (ACM), even when adjusted for common risk factors (14). This comes in line with other findings also supporting the correlation between specific tumour biomarkers and CVD outcomes (15, 16). Nowadays, most tumour biomarkers are known to have low sensitivity and specificity in diagnosing certain tumours. As a result, the role of tumour biomarkers is more significant in disease follow-up. This is potentially a direct consequence of their role in many pathways shared between different tumours and CVD. Some of the main mechanisms of cancer progression, such as abnormal cell division, increased metabolic activity, drug resistance, and immune-modulating signals have been established and recognised. Their counterparts: altered cell division and failure in tissue repair, ischemia, metabolic remodelling, energy deficit, and increased sensitivity to toxins, have been known to manifest in patients with CVD, especially HF. Thus, the shared pathophysiology between CVD and cancer could support the notion that many markers could be clinically relevant for both diseases.

However, the exact interplay between CVD and cancer and their similar pathophysiological milieus are still under investigation.

This study aims to evaluate the value of tumour biomarkers as predictors of CV outcomes in a general population. Hence, we conducted several analyses to evaluate whether tumour biomarkers can predict new-onset CVD and mortality in the Prevention of Renal and Vascular End-stage Disease (PREVEND) cohort.

## Materials and Methods

### Study Population

**Figure 1:**
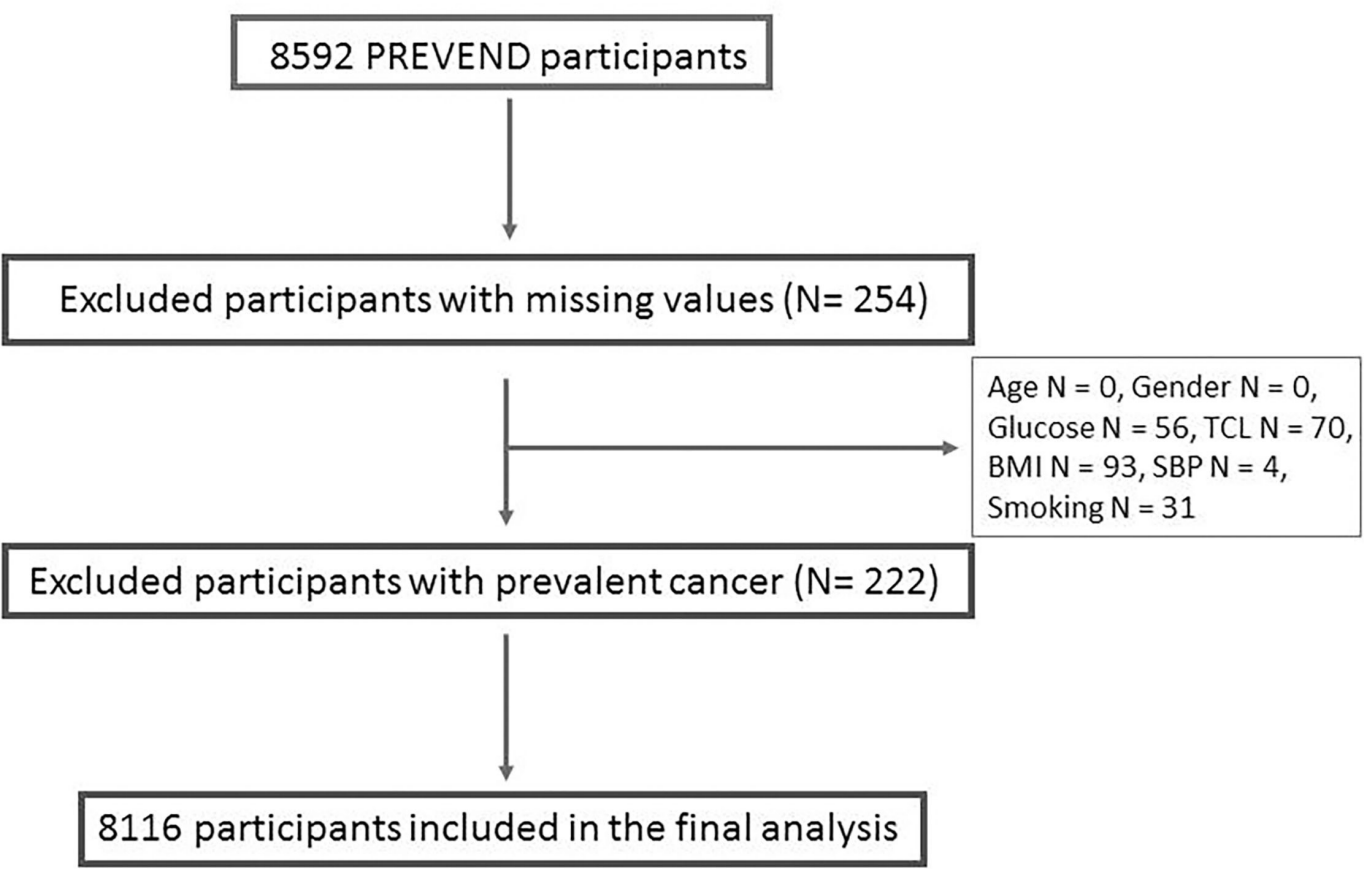
Detailed description of the participants excluded from the analysis. TCL, total cholesterol; BMI, body mass index; SBP, systolic blood pressure.

The PREVEND study is a prospective cohort that focused on renal and CV Diseases. Full details of recruitment methods have been reported elsewhere (17). In brief, from 1997 to 1998, all inhabitants of the city of Groningen, The Netherlands, aged 28 to 75 years (*n* = 85 421) were asked to send in a first-morning urine sample and complete a short questionnaire on demographics and CVD history. A total of 40 856 subjects responded (47.8%). All subjects with urinary albumin excretion (UAE) exceeding 10 mg/L (*n* = 7,786) as well as a randomly selected control group with a UAE 10 mg/L (*n* = 3,395) were invited to an outpatient clinic for a detailed assessment of CV and renal risk factors. After excluding subjects with insulin-dependent diabetes mellitus, pregnant women, and subjects unable or unwilling to participate, a total of 8,592 subjects completed the initial screening. For the current analysis, we've excluded subjects with prevalent cancer and missing variables used in our analysis, resulting in a total of 8,116 subjects (Figure 1). The PREVEND study was approved by the institutional medical Ethics Committee and conducted according to the Declaration of Helsinki. All subjects provided written informed consent. Baseline characteristics of the study population are presented in Table 1.

**Table 1:** Baseline characteristics of study population.

**Factor**	**Value**
*N*	8,116
Age (years), median (IQR)	48.1 (39.0, 59.7)
Gender, female (%)	4,067 (50.1%)
BMI, median (IQR)	25.5 (23.1, 28.3)
SBP (mmHg), median (IQR)	125 (114, 141)
Smoking (%)	3,083 (38.0%)
Cholesterol (mmol/L), median (IQR)	5.5 (4.8, 6.3)
Glucose (mmol/L), median (IQR)	4.7 (4.3, 5.1)
Prevalent MI (%)	483 (6.0)
Prevalent CVA (%)	71 (0.9)
Prevalent HF (%)	22 (0.3)
AFP (ng/mL), median (IQR)	2.7 (1.7, 4.1)
CA 125 (U/mL), median (IQR)	12.0 (8.6, 16.9)
CA 15–3 (U/mL), median (IQR)	17.1 (12.2, 22.5)
CA 19–9 (U/mL), median (IQR)	7.7 (5.0, 13.5)
CEA (ng/mL), median (IQR)	2.3 (1.6, 3.8)
CYFRA 21-1 (ng/mL), median (IQR)	1.5 (1.1, 2.1)
hsTnT (ng/L), median (IQR)	2.5 (2.5, 5)
NT-proBNP (ng/L), median (IQR)	37.3 (16.5, 73.3)

*BMI, body mass index; SBP, systolic blood pressure; MI, myocardial infarction; CVA, cerebrovascular accident; HF, heart failure; AFP, alpha-fetoprotein, CA, cancer antigen; CEA, carcinoembryonic antigen; CYFRA, cytokeratin fragment, hsTnT, high-sensitive troponine T; NT-proBNP, N-terminal pro-brain natriuretic peptide*.

### Study Parameters and Endpoints

For the study purposes, anthropometrics were measured, and blood and urine samples were collected. Subjects also filled in a detailed questionnaire, questions regarding family and own medical history were included. At least 10 blood pressure measurements were recorded for 10 min using an automatic Dinamap XL Model 9300 series device. Systolic blood pressure (SBP) was calculated as the mean of the last two measurements. Body mass index (BMI) was calculated as weight divided by height squared (kg/m^2^). Glucose, CRP and total cholesterol (TCL) were measured in collected blood samples. Active smoking was defined as current smoking or smoking within the previous year.

CV morbidity and mortality, and ACM were specified as primary endpoints, of which the association with six widely used biomarkers also known as “tumour biomarkers” was evaluated. In secondary analyses, we assessed the predictive significance of tumour biomarkers for CV mortality and specific CVDs, namely heart failure (HF), coronary artery disease (CAD) and cerebrovascular attack (CVA).

Incident CV morbidity was defined as CV event, while CV mortality was defined as death due to a CV event. CV events include acute myocardial infarction (MI), sub-acute and chronic CAD, haemorrhagic and ischemic CVA, and vascular interventions such as coronary artery bypass grafting (CABG), percutaneous coronary intervention (PCI), bypass grafting of the aorta, or peripheral vessels. Information on hospitalisation for CV event was retrieved from the Dutch national registry of hospital discharge diagnoses (PRISMANT). HF endpoint was adjudicated by a committee of HF experts and was separately included in the secondary endpoint. Time of follow-up was defined as the time between the baseline and CV event or death, up to 1 January 2011. Data were coded according to the International Classification of Diseases, Ninth Revision (ICD-9) and are described in detail elsewhere (18). ACM and CV mortality data were extracted from the Dutch Central Bureau of Statistics. If a person moved to an unknown destination, the date on which that individual could no longer be tracked *via* the municipal registry was used as the censor date (19).

### Tumour Biomarkers Assays

At baseline, EDTA plasma samples were collected from all participants and were stored at −80°C until the analysis. The following tumour biomarkers were measured: alpha- fetoprotein (AFP), cancer antigen 125 (CA 125), cancer antigen 15-3 (CA15-3), cancer antigen 19-9 (CA19-9), carcinoembryonic antigen (CEA), cytokeratin fragment 21-1 (CYFRA 21-1), and assessed. All six tumour biomarkers were measured by the Roche Elecsys assay on a Cobas E 411 analyser using standard methods (Roche Diagnostics GmbH, Mannheim, Germany) (14). This platform allows to quantitatively measure human AFP, CA 125, CA15-3, CA19-9, CEA and CYFRA 21-1 levels in plasma with high sensitivity. The number of participants with measured biomarkers is detailed in Table 2.

**Table 2:** Reference values of measured tumour biomarkers and their values in PREVEND population.

**Tumour biomarker**	**Malignancy**	**Reference**	**Mean**	***N***
		**value**	**value (SD)**	
AFP (ng/mL), median (IQR)	Germ cell	<15 or <8.4	3.6 (±13.7)	7,944
CA 125 (U/mL), median (IQR)	Ovarian	<35	14.3 (±10.9)	7,936
CA15-3 (U/mL), median (IQR)	Breast	<30	18.2 (±8.3)	7,941
CA19-9 (U/mL), median (IQR)	Pancreas	<37	10.9 (±16.4)	7,93
CEA (ng/mL), median (IQR)	Colon	<5	3.3 (±18.7)	7,946
CYFRA 21-1 (ng/mL), median (IQR)	Lung	<3.4	1.96 (±15.9)	7,919

*AFP, alpha-fetoprotein, CA, cancer antigen; CEA, carcinoembryonic antigen; CYFRA, cytokeratin fragment*.

### Statistical Analysis

As the PREVEND cohort has an overrepresentation of subjects with increased UAE, a statistical correction factor was employed using a weighted Cox regression model so that the conclusions may be extended to the general population, as previously published (19).

All data are presented as means and standard deviation (SD) when data were normally distributed, or as medians with interquartile ranges (IQR) when data were not normally distributed. Categorical variables are presented as percentages. Because of the skewed distribution, all biomarkers tested (AFP, CA 125, CA15-3, CA 19-9, CEA and CYFRA 21-1) were transformed to a log-scale. This means that risk estimates should be interpreted as a relative risk if values were doubled (e.g., 5–10 mg/L). Differences between two groups for normally distributed data were tested using two sample *t*-test while a Wilcoxon rank-sum test was used for non-normally distributed data. Cox proportional hazard regression analysis was performed for all end-points to evaluate the independent prognostic value of each tumour biomarker. For our primary and secondary analysis, the first model was adjusted for age and gender only. We accounted for type I error, so those variables that reached significance of *P*-value < 0.1 were included in the second multivariable (MV) analysis. We adjusted our MV model for age, gender, BMI, smoking status, SBP, plasma glucose, TCL levels, and prevalent CVD, in accordance with previously published results (19). Biomarkers reaching *P*-value < 0.05 were additionally corrected for C-Reactive Protein (CRP). Using the same MV correction model, we've tested sex-specific sensitivity of several biomarkers. Interpretation of the final results was done after performing Bonferroni type adjustment for multiple analyses, and a *p*-value of 0.008 (0.05/number of tests) was considered significant. Results are summarised as hazard ratios (HRs), with 95% confidence intervals based on robust standard error estimates. All statistical analyses and figures displaying HR's were made using STATA SE, version 14.2.

## Results

### Baseline Characteristics of Study Participants

The baseline characteristics of the participants are presented in Table 1. In summary, the median age of the study population was 48 years, 50% of the study population were females and overall CV risk was low. Participants were in general normotensive (median SBP 125 mmHg, IQR 114, 141 mmHg), non-diabetic (median glucose 4.7 mmol/L, IQR 4.3, 5.1 mmol/L), and not obese (median BMI 25.5 kg/m^2^, IQR 23.1, 28.3 kg/m^2^). Less than 39% of participants were active smokers and there were only 576 prevalent CV events recorded, of which 483 MI, 71 CVA and 22 HF events. The baseline median values of all six tumour biomarkers measured were not elevated. The reference mean values of each tumour biomarker were obtained from the previous literature and are presented in Table 2 (20–25).

### Tumour Biomarkers Predict CV Events and ACM

During the mean follow-up of 11.5 years, 920 participants experienced CV event defined as either CV morbidity or CV mortality, and total of 751 participants died (Supplementary Table 1). Levels of high-sensitive troponin T (hsTnT) and N-terminal pro-brain natriuretic peptide (NT-proBNP) were higher in participants with both prevalent and incident CVD, when compared to the participants without CVD, as reported in Table 3. However, five out of six tumour biomarkers levels (CA 125 being an exception) were significantly higher in participants with CVD when compared to the participants without CVD (*p* < 0.001).

**Table 3:** Cardiac and tumour biomarker levels in patients without and with CVD.

**Factor**	**No CVD (*N* = 7,222)**	**Incident CVD (*N* = 716)**	**Prevalent CVD (*N* = 178)**	***p*-value**
hs-cardiac troponin-T (ng/L), median (IQR)	2.5 (2.5, 4)	5 (2.5, 8)	7 (4, 10)	<0.001
N-terminal pro-BNP (ng/L), median (IQR)	34.8185 (15.695, 67.026)	55.262 (22.7585, 114.079)	183.601 (62.5, 400.719)	<0.001
AFP, median (IQR)	2.68 (1.74, 4.15)	2.875 (1.99, 4.23)	2.86 (2.1, 4.77)	<0.001
CA125, median (IQR)	12.01 (8.62, 16.94)	12.03 (8.88, 17.08)	12.49 (8.99, 18.16)	0.47
CA15-3, median (IQR)	16.75 (12.11, 22.22)	18.73 (13.84, 25.04)	19.01 (13.91, 24.23)	<0.001
CA19-9, median (IQR)	7.58 (4.96, 13.16)	8.54 (5.36, 15.69)	10.06 (6.18, 18.21)	<0.001
CEA, median (IQR)	2.28 (1.6, 3.71)	2.78 (1.94, 4.38)	3.19 (2.03, 4.91)	<0.001

*Hs-TnT, high-sensitive troponine T; NT-proBNP, N-terminal pro-brain natriuretic peptide; AFP, alpha-fetoprotein, CA, cancer antigen; CEA, carcinoembryonic antigen; CYFRA, cytokeratin fragment*.

Cox regression analysis shows a significant correlation between the levels of several tumour biomarkers and our primary endpoints, CV morbidity and mortality, and ACM (Tables 4–7). After multivariable (MV) adjustment for common risk factors and prevalent CVD, CEA presents as an independent predictor of CV events (HR 1.28, 95% CI 1.08–1.53), while CYFRA 21-1 did not reach the significance by a margin (HR 1.30, 95% CI 1.06–1.58). Even after further correction for inflammation (CRP), CEA remained a significant predictor of CV morbidity and mortality (HR 1.27, 95% CI 1.07–1.52). Two biomarkers including CA 15-3 and CEA revealed a strong correlation with ACM, even after full adjustment for our MV model and inflammation (HR 1.58, 95% CI 1.16–2.15; HR 1.60, 95% CI 1.30–1.98).

**Table 4:** Correlation of tumour biomarker levels, CV morbidity and mortality, and ACM.

	**Age/gender**		**MV correction**	
**CV**	**HR (95% CI)**	***P***	**HR (95% CI)**	***P***
CA 125	1.12 (0.94–1.34)	0.180		
CA 15-3	1.29 (1.02–1.63)	0.032	1.15 (0.91–1.42)	0.215
CA 19-9	1.09 (0.97–1.22)	0.126		
CEA	1.58 (1.36–1.83)	0.000	1.28 (1.08–1.53)	0.004
CYFRA 21-1	1.42 (1.19–1.70)	0.000	1.30 (1.06–1.58)	0.009
**ACM**	**HR (95% CI)**	***P***	**HR (95% CI)**	***P***
AFP	1.03 (0.88–1.22)	0.654		
CA 125	1.29 (1.05–1.58)	0.012	1.24 (1.01–1.53)	0.037
CA 15-3	1.51 (1.14–2.01)	0.004	1.58 (1.18–2.12)	0.002
CA 19-9	1.08 (0.95–1.23)	0.197		
CEA	1.78 (1.50–2.11)	0.000	1.60 (1.30–1.96)	0.000
CYFRA 21-1	1.38 (1.09–1.75)	0.007	1.24 (0.99–1.57)	0.060

*MV correction is adjusted for age, gender, BMI, smoking habits, TCL, glucose levels, SBP and prevalent CVD*.

*CV, cardiovascular; HR, hazard ratio; MV, multivariable; CVD, cardiovascular disease; ACM, all-cause mortality; BMI, body mass index; TCL, total cholesterol; SBP, systolic blood pressure; AFP, alpha-fetoprotein; CA, cancer antigen; CEA, carcinoembryonic antigen; CYFRA, cytokeratin fragment*.

**Table 5:** Correlation of tumour biomarker levels, CV morbidity and mortality, and ACM.

	**MV correction + CRP**	
**CV**	**HR (95% CI)**	***P***
CA 15-3	1.10 (0.87–1.40)	0.398
CEA 1.27	(1.07–1.52)	0.006
CYFRA 21-1	1.27 (1.03–1.56)	0.021
**ACM**	**HR (95% CI)**	***P***
CA 125	1.26 (1.01–1.56)	0.037
CA 15-3	1.58 (1.16–2.15)	0.003
CEA	1.60 (1.30–1.98)	0.000
CYFRA 21-1	1.24 (0.98–1.58)	0.065

*MV correction is adjusted for age, gender, BMI, smoking habits, TCL, glucose levels, SBP, prevalent CVD and CRP*.

*CV, cardiovascular; HR, hazard ratio; MV, multivariable; CVD, cardiovascular disease; ACM, all-cause mortality; BMI, body mass index; TCL, total cholesterol; SBP, systolic blood pressure; CA, cancer antigen; CEA, carcinoembryonic antigen; CYFRA, cytokeratin fragment*.

**Table 6:** Correlation of tumour biomarker levels and CV mortality, HF, CAD, and CVA.

	**Age/gender correction**		**MV correction**	
**CV mortality**	**HR (95% CI)**	***P***	**HR (95% CI)**	***P***
AFP	0.99 (0.73–1.35)	0.974		
CA 125	1.34 (0.91–1.97)	0.135		
CA 15-3	2.77 (1.56–4.92)	0.000	3.01 (1.70–5.32)	0.000
CA 19-9	1.22 (0.92–1.63)	0.160		
CEA	2.09 (1.64–2.66)	0.000	1.82 (1.30–2.56)	0.001
CYFRA 21-1	1.53 (1.05–2.23)	0.026	1.40 (0.94–2.10)	0.094
**HF**	**HR (95% CI)**	***P***	**HR (95% CI)**	***P***
AFP	0.95 (0.80–1.13)	0.625		
CA 125	1.19 (0.99–1.43)	0.063	1.28 (0.96–1.70)	0.086
CA 15-3	1.42 (1.10–1.83)	0.006	1.67 (1.15–2.42)	0.006
CA 19-9	1.12 (0.98–1.27)	0.072	1.21 (1.01–1.46)	0.037
CEA	1.31 (1.09–1.58)	0.004	1.31 (0.97–1.76)	0.071
CYFRA 21-1	1.17 (0.94–1.46)	0.147		
**CAD**	**HR (95% CI)**	***P***	**HR (95% CI)**	***P***
AFP	1.01 (0.84–1.22)	0.856		
CA 125	1.26 (1.01–1.56)	0.035	1.12 (0.89–1.41)	0.323
CA 15-3	1.21 (0.92–1.58)	0.158		
CA 19-9	1.11 (0.96–1.29)	0.131		
CEA	1.50 (1.25–1.81)	0.000	1.22 (0.99–1.51)	0.050
CYFRA 21-1	1.29 (1.03–1.61)	0.026	1,21 (0.94–1.55)	0.125
**CVA**	**HR (95% CI)**	***P***	**HR (95% CI)**	***P***
AFP	0.92 (0.67–1.20)	0.603		
CA 125	0.76 (0.55–1.05)	0.109		
CA 15-3	1.45 (0.84–2.51)	0.178		
CA 19-9	0.94 (0.76–1.17)	0.632		
CEA	1.50 (1.08–2.08)	0.015	1.22 (0.79–1.89)	0.362
CYFRA 21-1	1.79 (1.35–2.37)	0.000	1.53 (1.06–2.21)	0.020

*CV, cardiovascular; HR, hazard ratio; MV, multivariable; CVD, cardiovascular disease; HF, heart failure; CAD, coronary artery disease; CVA, cerebrovascular accident; BMI, body mass index; TCL, total cholesterol; SBP, systolic blood pressure; AFP, alpha-fetoprotein; CA, cancer antigen; CEA, carcinoembryonic antigen; CYFRA, cytokeratin fragment*.

*MV is adjusted for age, gender, BMI, smoking habits, TCL, glucose levels, SBP and prevalent CVD*.

**Table 7:** Correlation of tumour biomarker levels and CV mortality, HF, CAD, and CVA.

**CV mortality**	**HR (95% CI)**	***P***
CA 15-3	2.88 (1.58–5.24)	0.001
CEA	1.87 (1.33–2.64)	0.000
CYFRA 21-1	1.40 (0.93–2.13)	0.106
**HF**	**HR (95% CI)**	***P***
CA 125	1.27 (0.95–1.71)	0.097
CA 15-3	1.69 (1.16–2.48)	0.006
CA 19-9	1.22 (1.01–1.48)	0.036
CEA	1.28 (0.95–1.73)	0.096
**CAD**	**HR (95% CI)**	***P***
CA 125	1.11 (0.87–1.41)	0.389
CEA	1.21 (0.98–1.48)	0.070
CYFRA 21-1	1.18 (0.91–1.53)	0.200
**CVA**	**HR (95% CI)**	***P***
CYFRA 21-1	1.54 (1.06–2.26)	0.023

*CV, cardiovascular; HR, hazard ratio; MV, multivariable; CVD, cardiovascular disease; HF, heart failure; CAD, coronary artery disease; CVA, cerebrovascular accident; BMI, body mass index; TCL, total cholesterol; SBP, systolic blood pressure; AFP, alpha-fetoprotein, CA, cancer antigen; CEA, carcinoembryonic antigen; CYFRA, cytokeratin fragment*.

*MV is adjusted for age, gender, BMI, smoking habits, TCL, glucose levels, SBP, prevalent CVD and CRP*.

Furthermore, we conducted sex-specific analysis for the most prominent biomarkers, for our primary endpoints. Interestingly, while most CV events occurred in males (Supplementary Table 2) CYFRA 21-1 presented as a strong predictor of CV morbidity and mortality in females (HR 1.82, 95% CI 1.40–2.35), but not in males. CEA (HR 1.33, 95% CI 1.07–1.66) did not reach statistical significance for our CV model in males by a margin (Supplementary Table 3). The opposite was observed in ACM model, where only CEA predicted mortality in female gender (HR 1.64; 95% CI 1.20–2.24), while CEA and CYFRA 21-1 both show strong, independent correlation with ACM in male gender (HR 1.64, 95% CI 1.28–3.12; HR 1.55, 95% CI 1.18–2.02) (Supplementary Table 4).

### Correlation Between Tumour Biomarkers, CV Mortality and Specific CVDs

In our secondary analysis, we separately evaluated the predictive value of each tumour biomarker for CV mortality and specific CVDs. We focused namely on HF, CAD, and CVA events, as reported in Table 6. During a mean follow-up of 11.5 years, 215 participants died due to a CV event (Supplementary Table 1). After correction for our MV- inflammation model (age, gender, BMI, smoking, TCL, plasma glucose, SBP, prevalent CVD and CRP), two tumour biomarkers CA15-3 and CEA (HR 2.88, 95% CI 1.58–5.24 and HR 1.87, 95% CI 1.33–2.64) remained strongly associated with CV mortality (Table 7).

We also evaluated relevant predictors of HF, CAD and CVA. Three hundred and thirty seven participants developed HF, 612 participants of our study developed CAD and 222 developed CVA (Supplementary Table 1). While CA15-3 showed significant association with HF (HR 1.69, 95% CI 1.16–2.48), none of the biomarkers were significance for CAD and CVA (Tables 6, 7).

Lastly, we've divided most prominent biomarkers (CA 125, CA 15-3, CEA and CYFRA) in tertiles and evaluated the number of CVD event and time to events in each group (Supplementary Table 5). The tertile with the lowest value of biomarkers has also the lowest number of CVD events, while the time to the CVD event is longer. Tertile with highest value of all four biomarkers show highest number of CVD events and shortest time to the event.

## Discussion

**Figure 2:**
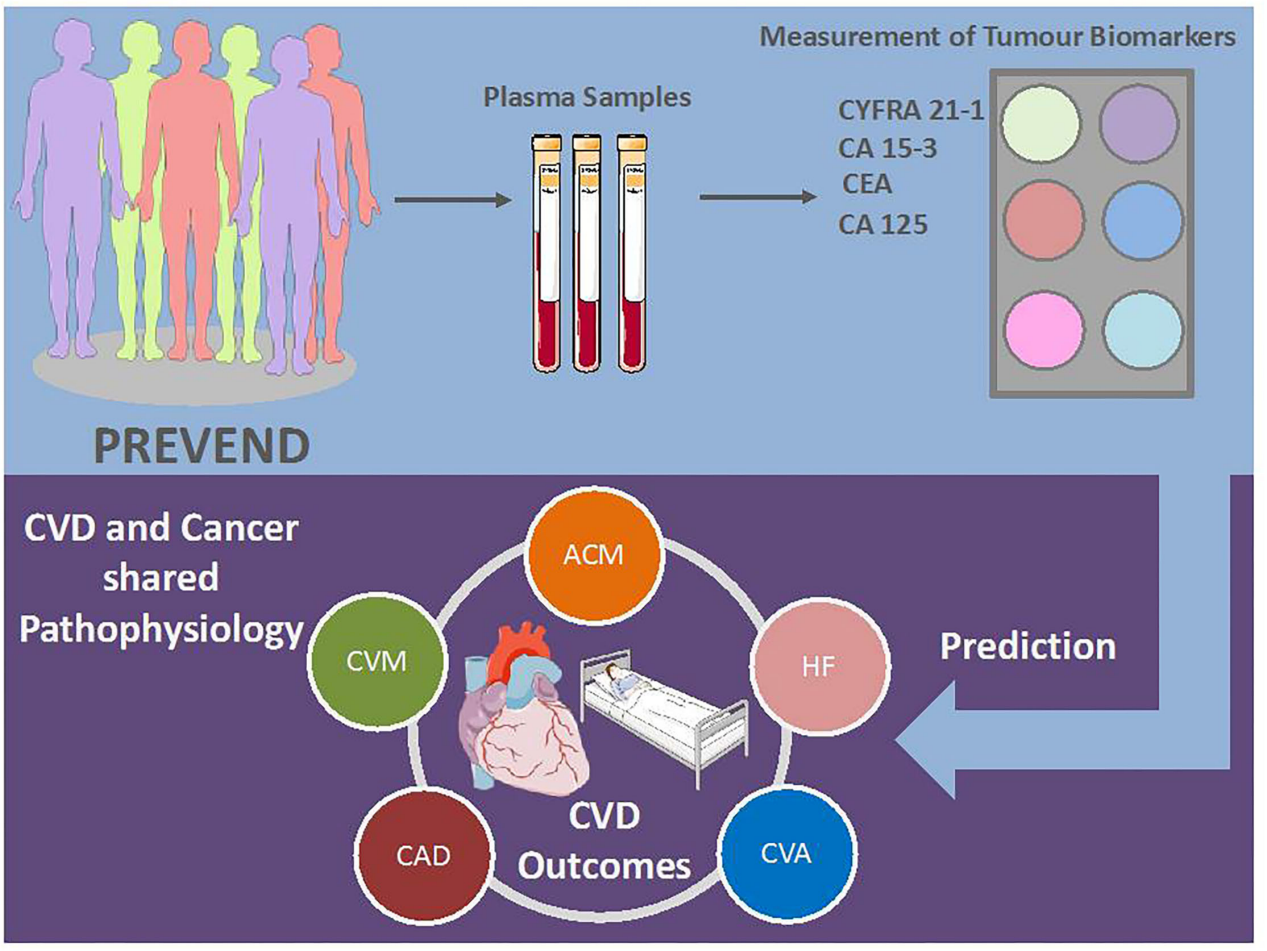
Possible use of tumour biomarkers in clinical practise. CVD, cardiovascular disease; AFP, alpha-fetoprotein; CA, cancer antigen; CEA, carcinoembryonic antigen; CYFRA, cytokeratin fragment; ACM; all-cause mortality; CVM, cardiovascular mortality; HF, heart failure; CAD, coronary artery disease; CVA, cerebrovascular accident. Illustration elements are from Smart Servier Medical Art.

A growing body of experimental and clinical evidence reveals numerous commonalities in the biology underlying both pathologies. However, the exact interplay between CVD and cancer is yet to be discovered. First, we demonstrated that CEA is a strong predictor of CV morbidity and mortality, and two (CA15-3 and CEA) of six tumour biomarkers are associated with ACM. Our secondary analysis showed that several tumour biomarkers are strongly associated with specific CVDs, even after the full adjustment for shared risk factors and prevalent CVD. These outcomes are indicative of shared pathophysiologic mechanisms and connections between cancer and CVD (Figure 2). Moreover, our data reveal that specific pathological mechanisms leading to different CVD are also involved in cancer. Furthermore, the predictive value of tumour biomarkers is sex specific.

### The Association Between Tumour Biomarkers and CVD

Tumour biomarkers are in use for early detection of cancer and monitoring tumour progression of diagnosed cancer. When evaluating the possible mechanisms linking tumorigenesis and CVD, understanding the biomarker background becomes a vital piece of the puzzle.

CEA is a surface glycoprotein mainly found in epithelial and mucus-secreting cells of the colon and is involved in cancer invasion and metastasis. It plays an important diagnostic and prognostic role in colorectal cancer. CEA plays a crucial role in cell adhesion and metastatic dissemination of intestinal cancer by activating cytokine cascade through direct binding with monocytes (26, 27). The same immunological mechanisms could explain the link between tumour biomarkers and CVD. A recent study suggests that elevated CEA levels correlate with elevated leukocytes count, suggesting the relation between CEA and chronic inflammation (28). Inflammation cells, monocytes and cytokines they release (such as TNF-α, IL-1β, IL-6) have also been found to play an important role in different cardiovascular pathological processes, such as atherosclerosis, myocardial infarction, myocardial remodelling and heart failure, and arrhythmias (29–31). We've previously shown that CEA predicts ACM in patients with severe HF (14). Our results reveal, for the first time, CEA as a potential predictor of incident CV events and ACM (Tables 4, 5). Furthermore, the secondary analysis suggested that CEA also independently predicts CV mortality (Tables 6, 7).

CA15-3 is a soluble form of mucin 1 (MUC1), trans-membrane glycoprotein first recognised as a prognostic biomarker in breast cancer. MUC1 has been found on mucosal surfaces of many different organs. Therefore, it is expected that elevated levels of CA15-3 have also been found in some non-malignant conditions, including acute MI (32). Based on this data it has been suggested that CEA could potentially be a marker of destruction and pathological leakage of epithelial cells, and their products into the blood (32). Furthermore, MUC1 is known as a natural ligand for galactin-3, a suspected miscreant protein responsible for, among others, organ fibrosis, atherosclerosis, and HF (33, 34). We have previously reported that CA15-3 is strongly correlated with HF severity and ACM in the HF population (14). The current results comply with the studies, as CA15-3 presents as an strong and independent biomarker of ACM and CV mortality (Tables 4–7). While we did not find a direct correlation between CA15-3 and CAD, our study still indirectly supports previously mentioned results (32), as we do show that CA15-3 strongly correlates with CV mortality.

CYFRA 21-1 is a measurement of cytokeratin 19 (CK19) fragment, also known as a pan-carcinoma marker. It is used as a prognostic marker in over 30 different types of cancer (35). It is postulated that the elevated levels of this biomarker are a result of abnormal cellular mitosis and apoptosis, processes often seen in both carcinogenesis and CVD. However, the exact mechanisms and role of CYFRA 21-1 in tumour growth and dissemination are still unknown (35). This is the first time elevated CYFRA 21-1 levels have been linked to CVD risk in healthy individuals. Previously, we have shown a significant correlation between CYFRA 21-1 and ACM in the HF population (14). In the current study, we did not observe the same trend for HF. This could be attributed to the difference in male and female proportions between two studies (74% of HF population were males in the previous study). Furthermore, the new analyses suggest that CYFRA 21-1 could be sex specific since it consistently and independently predicted new CV events in female, and not in male patients (Supplementary Tables 3, 4).

The only tumour biomarker that has been previously studied in CVD was CA 125. Elevated levels of CA125 are frequently detected in numerous malignancies, such as lung, breast, colon, as well as in non-malignant situations, which makes it less specific. Nevertheless, CA 125 is reported as an important prognostic biomarker in HF, CAD, and atrial fibrillation (15, 16, 22, 36). In this study, CA 125 did not show a statistically significant correlation with either primary or secondary endpoints (Tables 4–7). Our results suggest that CA 125 levels may be associative with the disease severity and its role is therefore less significant in healthy population.

Lastly, the two tumour biomarkers AFP and CA19-9 did not show any correlation with the primary and secondary outcomes. The negative correlation with CVD prevalence is in accordance with the previous work (14). While CA19-9 levels seem to correlate some CV risk factors, especially glucose and HbA1c levels in diabetic and pre-diabetic individuals (37, 38), our data imply that there is no direct pathophysiological link between CA19-9 and both CV events and ACM (Tables 4–7).

### Study Strengths and Limitations

Our analyses are performed in a good-sized prospective cohort. Several biomarkers significantly predicted CV outcomes and mortality, and specific CVD events, like HF. This supports the substantial commonality between cancer and CVD. Moreover, half of the participants were women. Lastly, our analysis excluded subjects with a known history of cancer, making the tumour biomarker levels presentable for the general population.

The PREVEND cohort was enriched for increased albumin excretion. Thus, this overexpression was compensated by applying statistical correction. The cut-off points are predominantly used for cancer diagnosis and may differ for CVD diagnosis. We cannot disregard the possibility that a number of subjects with cancer could have remained unrecognised and still included in the final analysis. Furthermore, the PREVEND cohort is predominantly a European Caucasian cohort, which makes it difficult to generalise the outcomes to other ethnicities and populations. Our population has a relatively low number of CV events. The current study is observational; thus, we cannot draw any conclusions regarding the causality. Considering the fact that cancer and CVD are general terms that refer to large groups of heterogeneous diseases, pre-clinical models of cancer and CVDs are needed to assess the mechanisms behind the production of biomarkers in different diseases aetiologies. Furthermore, available biobanks from patients with cancer or CVDs could be used for omics analyses to evaluate the global changes at the molecular level, and to identify new common biomarkers.

### Clinical Implications

By showing a strong, independent link between tumour biomarkers, CVD and ACM, we further support the notion that cancer and CVD share more than just common risk factors. We encourage clinicians to consider the strong association between these two disease entities to improve patient's management. In-depth knowledge of specific pathophysiological mechanisms involving both syndromes, could lead to a better prevention, earlier diagnosis, personalised treatments and improved quality of life of both cancer and CV patients.

Our study demonstrates a strong correlation between several tumour biomarkers and new-onset CVD, all-cause, and CV mortality in a presumably healthy population. This suggests that the operative pathways in cancer mediating the production and release of tumour biomarkers are also present in CVD. The outcomes of the current study suggests that the assessed tumour biomarkers could be useful for risk stratification of cancer patients that are prone to develop incident CV events. Validating these results in other cohorts, and at the molecular level are essential before considering these biomarkers for clinical use as predictors of CV outcomes and mortality.

We, therefore, encourage further research for the use of tumour biomarkers in the prognosis and evaluation of CVD.

## Data Availability Statement

Raw data may be made available upon reasonable request.

## Ethics Statement

The studies involving human participants were reviewed and approved by institutional medical Ethics Committee. The patients/participants provided their written informed consent to participate in this study.

## Author Contributions

RB, VB, and JPA designed the study. VB set up the database and did the statistical analyses. NS helped with the statistical analyses. VB and JPA co-drafted the first version of the article. VB and CS performed the biomarkers measurements. RB, NS, SW, WM, IK, and CS provided critical revision of the manuscript. All authors interpreted the results and critically revised the manuscript for scientific content and approved the final version of the article.

## Funding

This work was supported by a grant from the European Research Council (ERC CoG 818715, SECRETE-HF). Furthermore, support was received from grants from the Netherlands Heart Foundation (CVON SHE-PREDICTS-HF, grant 2017-21; CVON RED-CVD, grant 2017-11; CVON PREDICT2, grant 2018-30; and CVON DOUBLE DOSE, grant 2020B005), and by a grant from the leDucq Foundation [Cure PhosphoLambaN induced Cardiomyopathy (Cure-PLaN)].

## Conflict of Interest

The UMCG, which employs several of the authors, has received research grants and/or fees from AstraZeneca, Abbott, Bristol-Myers Squibb, Novartis, Novo Nordisk, and Roche. RB received speaker fees from Abbott, AstraZeneca, Bayer, Novartis, and Roche. The remaining authors declare that the research was conducted in the absence of any commercial or financial relationships that could be construed as a potential conflict of interest.

## Publisher's Note

All claims expressed in this article are solely those of the authors and do not necessarily represent those of their affiliated organizations, or those of the publisher, the editors and the reviewers. Any product that may be evaluated in this article, or claim that may be made by its manufacturer, is not guaranteed or endorsed by the publisher.
