# Supplementary material for: Established Tumour Biomarkers Predict Cardiovascular Events and Mortality in the General Population
Source: Front Cardiovasc Med. 2021 Dec 8;8:753885. doi: 10.3389/fcvm.2021.753885 (PMC8692719; doi:10.3389/fcvm.2021.753885)
Supplement: Supplementary file 1 [file Data_Sheet_1.docx]

Supplement Table 1. Incident CV events in study population.

| **Incident events** | **N** | **%** |
| --- | --- | --- |
| CAD | 612 | 7.55 |
| CVA | 222 | 2.74 |
| HF | 337 | 4.15 |
| ACM | 751 | 9.25 |
| CV mortality | 215 | 2.65 |
| Cancer | 834 | 10.28 |

CV, cardiovascular; CAD, coronary artery disease; CVA, cerebrovascular accident; HF, heart failure; ACM, all-cause mortality.

Supplement table 2. Sex-specific incidence of cardiovascular events.

| **Factor** | **Female** | **%** | **Male** | **%** |
| --- | --- | --- | --- | --- |
| N | 4076 |  | 4049 |  |
| CV total | 278 | 6.84 | 642 | 15.8 |
| CAD | 170 | 4.18 | 442 | 10.9 |
| CVA | 84 | 2.07 | 138 | 3.41 |
| HF | 119 | 2.93 | 218 | 5.3 |
| Prevalent HF | 1 |  | 21 | 0.5 |
| Prevalent MI | 167 | 4.19 | 316 | 7.9 |
| Prevalent CVA | 30 | 0.75 | 41 | 1.04 |
| Cancer | 751 | 18.47 | 913 | 22.5 |
| ACM | 239 | 5.8 | 512 | 12.6 |
| CVM | 59 | 1.4 | 156 | 3.8 |

CV, cardiovascular; CAD, coronary artery disease; CVA cerebrovascular attack; HF, heart failure; MI, myocardial infaction; ACM, all-cause mortality; CVM, cardiovascular mortality.

Supplement table 3. Correlation of tumour biomarker levels, CV morbidity and mortality and gender. MV correction is adjusted for age, BMI, smoking habits, TCL, glucose levels, SBP and prevalent CVD.

| *FEMALE* | **Age/gender correction** |  | **MV correction** |  |
| --- | --- | --- | --- | --- |
|  | **HR (95% CI)** | **P** | **HR (95% CI)** | **P** |
| **AFP** | 0.84 (0.66 - 1.06) | 0.153 |  |  |
| **CA 125** | 1.09 (0.79 - 1.49) | 0.582 |  |  |
| **CA 15-3** | 1.42 (0.92 - 2.19) | 0.104 |  |  |
| **CA 19-9** | 1.11 (0.90 - 1.36) | 0.296 |  |  |
| **CEA** | 1.53 (1.22 - 1.92) | 0.000 | 1.27 (0.96 - 1.67) | 0.086 |
| **CYFRA 21-1** | 1.85 (1.44 - 2.38) | 0.000 | 1.82 (1.40 - 2.35) | 0.000 |
| *MALE* | **Age/gender correction** |  | **MV correction** |  |
|  | **HR (95% CI)** | **P** | **HR (95% CI)** | **P** |
| **AFP** | 1.03 (0.84 - 1.25) | 0.756 |  |  |
| **CA 125** | 1.15 (0.93 - 1.42) | 0.188 |  |  |
| **CA 15-3** | 1.23 (0.93 - 1.62) | 0.140 |  |  |
| **CA 19-9** | 1.08 (0.94 - 1.24) | 0.247 |  |  |
| **CEA** | 1.61 (1.32 - 1.95) | 0.000 | 1.33 (1.07 - 1.66) | 0.010 |
| **CYFRA 21-1** | 1.20 (0.96 - 1.50) | 0.097 |  |  |

CV, cardiovascular; HR, hazard ratio; MV, multivariable; CVD, cardiovascular disease; ACM, all-cause mortality; BMI, body mass index; TCL, total cholesterol; SBP, systolic blood pressure; AFP, alpha-fetoprotein, CA, cancer antigen; CEA, carcinoembryonic antigen; CYFRA, cytokeratin fragment.

Supplement table 4. Correlation of tumour biomarker levels, ACM and gender. MV correction is adjusted for age, gender, BMI, smoking habits, TCL, glucose levels, SBP and prevalent CVD.

| *FEMALE* | **Age/gender correction** |  | **MV correction** |  |
| --- | --- | --- | --- | --- |
|  | **HR (95% CI)** | **P** | **HR (95% CI)** | **P** |
| **AFP** | 0.90 (0.70 - 1.16) | 0.434 |  |  |
| **CA 125** | 1.14 (0.80 - 1.62) | 0.452 |  |  |
| **CA 15-3** | 1.80 (1.10 - 2.95) | 0.019 | 1.83 (1.08 - 3.09) | 0.023 |
| **CA 19-9** | 1.15 (0.95 - 1.41) | 0.143 |  |  |
| **CEA** | 1.76 (1.36 - 2.27) | 0.000 | 1.64 (1.20 - 2.24) | 0.002 |
| **CYFRA 21-1** | 1.08 (0.75 - 1.56) | 0.656 |  |  |
| *MALE* | **Age/gender correction** |  | **MV correction** |  |
|  | **HR (95% CI)** | **P** | **HR (95% CI)** | **P** |
| **AFP** | 1.16 (0.95 - 1.42) | 0.133 |  |  |
| **CA 125** | 1.34 (1.05 - 1.72) | 0.018 | 1.38 (1.07 - 1.78) | 0.012 |
| **CA 15-3** | 1.35 (0.95 - 1.91) | 0.091 | 1.31 (0.91 - 1.88) | 0.139 |
| **CA 19-9** | 1.04 (0.88 - 1.22) | 0.628 |  |  |
| **CEA** | 1.81 (1.45 - 2.27) | 0.000 | 1.64 (1.28 - 3.12) | 0.000 |
| **CYFRA 21-1** | 1.65 (1.23 - 2.20) | 0.001 | 1.55 (1.18 - 2.02) | 0.001 |

CV, cardiovascular; HR, hazard ratio; MV, multivariable; CVD, cardiovascular disease; ACM, all-cause mortality; BMI, body mass index; TCL, total cholesterol; SBP, systolic blood pressure; AFP, alpha-fetoprotein, CA, cancer antigen; CEA, carcinoembryonic antigen; CYFRA, cytokeratin fragment.

Supplement table 5. Incident CVD events by biomarker levels.

| **Tertiles CEA** | N | N CVD events | Mean T to CVD event (SD) |
| --- | --- | --- | --- |
| 1 | 2682 | 196 | 4098.9 (1159.1) |
| 2 | 22623 | 324 | 3984.1 (1244.4) |
| 3 | 2641 | 415 | 3911.3 (1348.2) |
| **Tertiles CA15-3** |  |  |  |
| 1 | 2647 | 223 | 4053.4 (1207.0) |
| 2 | 2647 | 305 | 3988.8 (1276.6) |
| 3 | 2647 | 376 | 3939.1 (1295.3) |
| **Tertiles CYFRA 21-1** |  |  |  |
| 1 | 2640 | 196 | 4089.8 (1162.8) |
| 2 | 2656 | 307 | 4030.5 (1227.7) |
| 3 | 2623 | 398 | 3859.9 (1374.0) |

CVD, cardiovascular; CEA, carcinoembryonic antigen, N, number; T, time; SD, standard deviation; CA15-3, cancer antigen 15-3, CYFRA 21-1, cytokeratin fragment 21-1
